# Experimental Study on the Coupling Mechanism of Sensors under a Strong Electromagnetic Pulse

**DOI:** 10.3390/s22145357

**Published:** 2022-07-18

**Authors:** Jie Cao, Minxiang Wei, Dong Zhou

**Affiliations:** 1 College of Energy and Power Engineering, Nanjing University of Aeronautics and Astronautics, Nanjing 210016, China; minxiangfwei@nuaa.edu.cn; 2 College of Field Engineering, Army Engineering University of PLA, Nanjing 210000, China; dongzhouv587@163.com

**Keywords:** main fuel regulation control system, pulse current injection, speed sensor, linear variable differential transformer

## Abstract

This paper presents a study on the electromagnetic effects of a main fuel control system under the action of a strong electromagnetic pulse. It assesses the electromagnetic sensitivity of a speed sensor and a linear variable differential transformer (LVDT) sensor. This assessment focuses on the control system’s electromagnetic effects and the sensors’ coupling signals. The effects and signals were determined using radiation and pulse current injection tests. Analysis of the signal at the system power port shows that it is the same as those for the two test methods. Based on analysis of the working mechanism and terminal signals of the sensors, the electromagnetic sensitivity of the system is different under the different electromagnetic pulse conditions. The electromagnetic sensitivity characteristics of the sensors were verified, and the electromagnetic effects of the main fuel regulation control system were analyzed. Meanwhile, the degree of sensor coupling mechanism caused by the electromagnetic coupling effects of the main fuel regulation control system under strong electromagnetic pulses were studied. These findings have clear practical implications for electromagnetic pulse protection of aero-engine control systems.

## 1. Introduction

Electronic devices and circuits are prone to generate a large pulse current that is more than 1 A or a high voltage which is more than 100 V under the impact of an electromagnetic pulse [1], resulting in erroneous control system instructions and incorrect operation of the actuator, affecting the realization of its function. This is because the control unit controls the actuator through switching devices, which mainly include solenoid valves, relays, power diodes, and field-effect transistors. The electromagnetic pulse burns the power switch, disturbing the normal motor rotation and switch action, causing the electromagnetic effect of the actuator. Electromagnetic pulse energy destroys integrated chips and semiconductor devices, resulting in the loss of data transmission, restarting equipment, and even burning integrated circuits, resulting in permanent damage to equipment [2]. In the current military and civilian fields, the power used by numerous radars, communication equipment, signal base stations, and other radiation equipment has increased. The electromagnetic pulses generated by natural electromagnetic sources such as lightning and electrostatic discharge have also increased, and the electromagnetic environment in the limited area where an electronic system is located becomes complex. There is no doubt that the degree of electrification of electronic systems is proportional to their sensitivity in complex electromagnetic environments. The higher the degree of electrification of the device, the more sensitive it will be [3,4,5].

**Figure 1:**
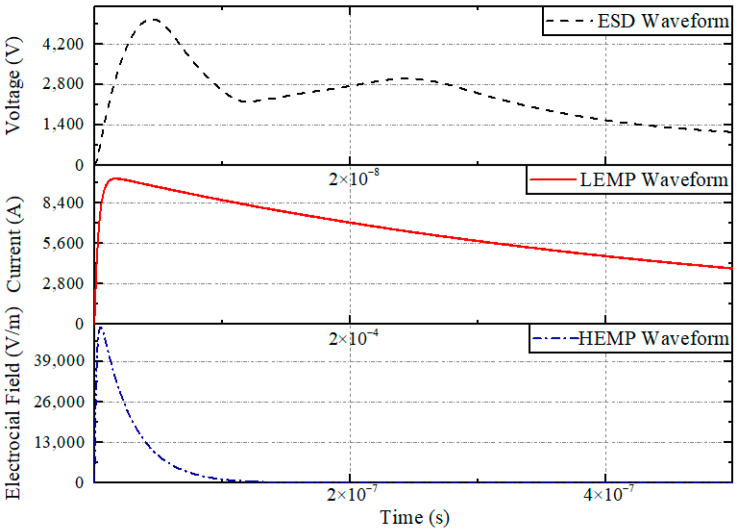
Familiar electromagnetic pulses.

Familiar electromagnetic pulses include lightning electromagnetic pulses (LEMPs), electro-static discharge (ESD), and high-altitude nuclear electromagnetic pulse (HEMP). These pulses have the characteristics of a steep front, high peak value, and wide frequency band, as shown in Figure 1. The electromagnetic pulse is coupled into a control system, resulting in logical errors in a system’s processing and even damage to the hardware of the controller [6]. However, the existing research on the sensitivity of aero-engine main fuel regulation control systems under the action of strong electromagnetic pulses is limited, and more research is required to support their quantitative analysis. Usually, the ability of equipment under test (EUT) to inevitably degrade performance under various electromagnetic interference conditions is called sensitivity, i.e., equipment or system performance degradation occurs when intentional electromagnetic interference occurs. Based on the theory of electromagnetic field, the characteristics of the electromagnetic environment are simulated to realize the equivalent substitution of the real environment and ensure the reliability of the test. In addition, the electromagnetic effects of the equipment under laboratory conditions are the same as in real-world scenarios.

With respect to the problem of strong electromagnetic pulse sensitivity of electronic control systems, two methods are normally used for analysis and study. The numerical calculation method establishes the electrical model of the control system to study and analyze it. However, due to the complexity of the structure and the nonlinearity of the control strategy for the main fuel regulation control system, it is often problematic to accurately complete the electronic model. Through the test method, the electromagnetic sensitivity effect of the control system can be observed intuitively; however, the test conditions are highly desirable. In general, the numerical calculation method can greatly improve the efficiency of analysis, but the accuracy depends on precise parameter modeling. The experimental test method can directly test the electromagnetic sensitivity of the system, but it needs to build an experimental platform. Compared with the numerical calculation method, the experimental method can better simulate the electromagnetic characteristics of the control system in a real electromagnetic environment.

There are two common sensitivity test methods: the radiation test method and the injection test method. The radiation test method tests the electromagnetic pulse effect of EUT through the radiation antenna and tests its coupling path under the action of a force electromagnetic pulse, which provides a reference for injection tests and protection verification. Because the radiation test needs to apply a high source excitation voltage at the radiation antenna end, the test process must ensure sufficient safety distance. Usually, test personnel stay in the anechoic chamber to monitor and record the results. At the same time, the radiation source needs to be discharged after each test. G. Lubkowski studied the response of UAV sensor systems under the attack of strong electromagnetic pulses [7]. The main electromagnetic effect caused by the pulses is interference with the engine’s control signal. The speed of the UAV motors shows rapid variations, caused by the disturbance effects observed in the UAV accelerometer. Longying Guo studied the electromagnetic pulse effect of a vehicle engine outside the cabin and established an accurate electromagnetic pulse test system [8]. The pulsed coupling signals in fuel injection are the main reason for outboard engine breakdown. The injection test method conducts electromagnetic pulse effect tests on EUT through direct or indirect injection methods to test the sensitivity of the equipment. Based on the radiation test method, the main fuel regulation control system is tested by the pulse current injection method, which solves the problem of a long-distance interconnected line not reaching the maximal threat state in the limited space of an anechoic chamber and verifies the interference law of the control system under the action of a strong electromagnetic pulse [9,10,11]. Zhou researched the main electromagnetic pulse coupling path of the main fuel regulation control system is the cable [12].

Reference [6] analyzed the electromagnetic sensitivity of the UAV sensors system utilizing the radiation test. The electromagnetic sensitivity of the engine was studied by the radiation test method, and the interference of electromagnetic pulse on the engine injection system was obtained [8]. Reference [13] shows that the data link system, as the hardcore unmanned aerial vehicle, is susceptible to strong electromagnetic pulse interference. Front door coupling is the main coupling path for the interference to unmanned aerial vehicles’ main remote control data link. Reference [14] indicates that the engine control system is susceptible to the electromagnetic pulse, making the control signal confused, and it can even result in the suspension of work. Reference [15] studied the electromagnetic disturbance of mobile system memory I/O buffer by the injection test method; it helps improve immunity over a certain frequency range. There is less research literature on the degrees of sensors’ coupling mechanisms by electromagnetic coupling effects of the main fuel control system under a strong electromagnetic pulse. Accordingly, that will be researched in this paper.

Many studies have researched the influence of the structure and external electromagnetic environment interference on the sensor [16,17,18], but these studies are all analyses of sensors and have certain limitations. Since sensors are often applied to control systems, the separate analysis of sensors is not particularly important. In addition, when a strong electromagnetic pulse enters the electronic equipment through the sensor, the control system will not fully produce an electromagnetic effect. In this paper, regarding the sensitivity effect on an aero-engine digital control system under the action of a strong electromagnetic pulse, the electromagnetic pulse radiation test and the pulse current injection test method are used to solve the above problems. After an electromagnetic pulse enters the sensitive device, it can make the electromagnetic coupling effect of the aero-engine digital control system. It provides a basis for the protection design of the control system.

In this section, coupling research between electromagnetic pulses and electronic systems in recent years has been briefly described. In Section 2, the electromagnetic pulse sensitivity test of the aero-engine digital controller is carried out by an electromagnetic pulse radiation test and pulse current injection test, and the corresponding signal of the controller is obtained. Section 3 analyzes the test results to obtain the electromagnetic coupling effect of the control system. Section 4 summarizes the paper.

## 2. Experiment

In this paper, two test methods were used to investigate the sensitivity effect on the main fuel regulation control system of aero-engines under the action of a strong electromagnetic pulse, and the sensitivity effect was determined.

**Figure 2:**
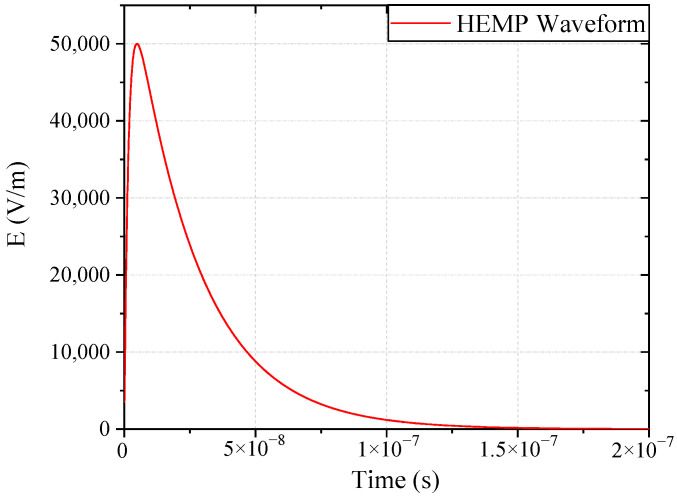
Time domain waveform of HEMP (E1 period).

**Figure 3:**
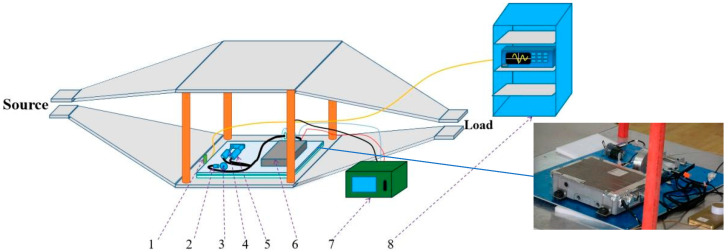
Main fuel regulation control system in the radiation test: 1—Field monitoring probe; 2—Speed sensor; 3—Phonic wheel; 4—LVDT; 5—Linear motor; 6—Controller; 7—Shielding box; 8—Shielding cabinet.

**Figure 4:**
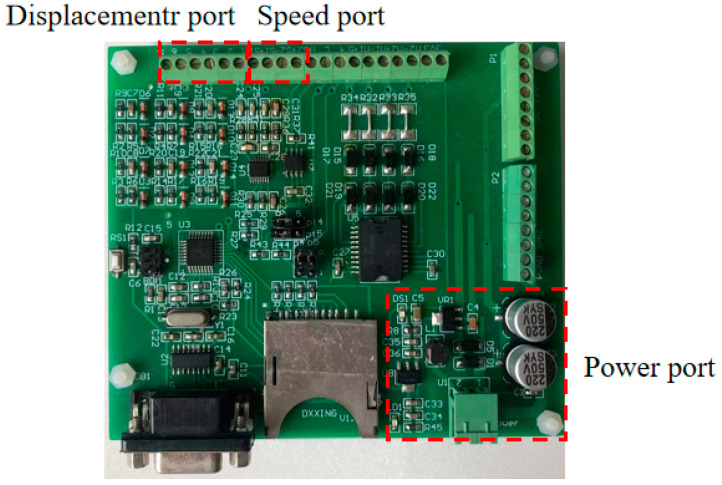
Main fuel regulation control system in the radiation test.

The radiation test places the main fuel regulation control system in the electromagnetic pulse radiation field to study the interference and damage under electromagnetic pulse radiation. The high-altitude nuclear electromagnetic pulse (HEMP) can be approximated as a plane wave near the ground. In this study, we used a transverse electromagnetic (TEM) chamber to test the control system, to simulate the high-altitude nuclear electromagnetic effect on near-ground facilities or equipment. The electromagnetic pulse radiation sensitivity effect test was carried out on the system according to the MIL-STD-461F RS105: Radiated Susceptibility, Transient Electromagnetic Field, or EMP (Electromagnetic Pulse) [19]. The RS105 test method describes a transient electromagnetic pulse of up to 50 kV/m, a double exponential waveform with a rise time in the nanosecond range, which is applied to the EUT at least 5 times. The pulse signal is shown in Figure 2. The mathematical expression of the pulse signal is shown in (1), where *E* and *E*_0_ are electric field strengths; *k*, *α*, and *β* are waveform factor, attenuation coefficient, and phase shift coefficient of HEMP. The layout of the main fuel control system in the radiation test is shown in Figure 3. During the test, the printed circuit board (PCB) port signal of the controller was monitored, including the coupling signal of speed, displacement, and power port, as shown in Figure 4.

*E* = *E*_0_*k*(*e*^−*αt*^ − *e*^−*βt*^), (1)

Through the time and frequency domain analysis of the monitoring port signals, the peak value and resonant frequency of the coupling signal of the aero-engine’s main fuel regulation control system—under the action of a strong electromagnetic pulse—can be determined. At the same time, the radiation test results can provide a basis for the pulse current injection test.

The injection test injects a voltage or current signal into the main fuel regulation control system to test its sensitivity. The electromagnetic sensitivity of the control system was obtained by selecting different injection frequencies to inject pulse current signals into the signal cables and power lines that are attached to the control system connector. Based on the monitored results of the controller port in the radiation test, the coupling voltage and current signal at the main fuel controller’s internal port, under the action of a strong electromagnetic pulse, is essentially a damping attenuation signal. Therefore, the damping sinusoidal current signal can be injected to simulate the electromagnetic effect of radiation from a strong electromagnetic pulse.

**Figure 5:**
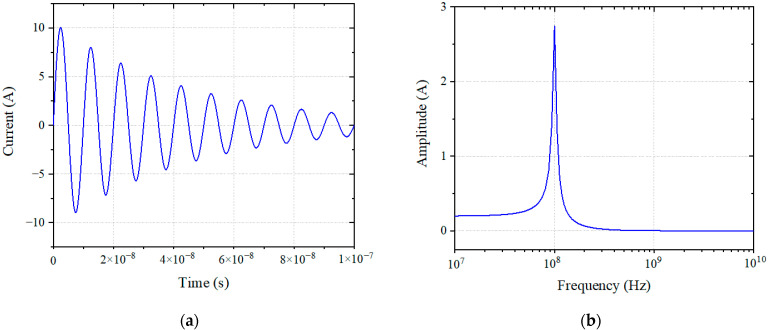
Pulse current injection signal: (**a**) time domain signal; (**b**) frequency domain signal.

**Figure 6:**
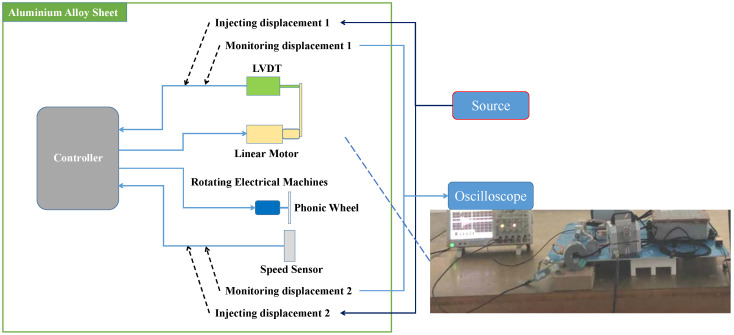
Main fuel regulation control system in the injection test.

During the pulse current injection test, the coupling voltage signals of the speed sensor and LVDT sensor port were monitored. By adjusting the output level of the pulse signal source, the measured peak current was recorded, and the performance of the system was monitored. A pulse current signal at 100 MHz is shown in Figure 4. In the experiment, the frequencies selected for the injected signal were 10 kHz, 100 kHz, 1 MHz, 10 MHz, 30 MHz, and 100 MHz. According to the damping sinusoidal pulse current conduction sensitivity test in MIL-STD-461F CS116 for the control system [14], this produces the sensitivity effect of the system under a strong electromagnetic pulse. The mathematical expression of the pulse signal is shown in (2), where *I* and *I_m_* are injection current strength, and *f* and *σ* are the frequency and damping coefficient of the injecting current signal. When the frequency of the pulse current signal changes, the period and the center frequency of the pulse signal change. The period of the time the domain signal is the reciprocal of frequency, and the center frequency of the frequency domain signal is the pulse current signal frequency. A pulse current signal at 100 MHz is shown in Figure 5. In the experiment, the frequencies selected for the injected signal were 10 kHz, 100 kHz, 1 MHz, 10 MHz, 30 MHz, and 100 MHz. According to the damping sinusoidal pulse current conduction sensitivity test in MIL-STD-461F CS116 for the control system [19], this produces the sensitivity effect of the system under a strong electromagnetic pulse. The layout of the main fuel control system in the injection test is shown in Figure 6.

*I* = *I_m_* sin(2*πft*) *e*^−*σt*^, (2)

By monitoring the signals and phenomena, the sensitivity thresholds of the sensors could be obtained, which provided a reference for electromagnetic pulse protection. The parameters of (1) and (2) are listed in Table 1.

By comparing the signal of the controller under the two test methods, the two are equivalent to the controller terminal under the electromagnetic pulse effect test. When the pulse current is injected into the main fuel regulation control system, the coupling effect of the system can be obtained by monitoring the differential mode voltage of the controller PCB ports, as well as monitoring the sensitivity phenomenon and sensitivity threshold of the control system, which provides support for electromagnetic protection and reinforcement of the system.

## 3. Results and Analysis

In this study, the coupling signals of the power port of the main fuel regulation control system were selected for comparison, and the radiation and injection test results were analyzed to prove the electromagnetic equivalence of the two test methods to the controller. By comparing the coupling signals of the speed sensor and the linear variable differential transformer sensor under the action of a strong electromagnetic pulse, the differences and commonalities of the sensors in the process of electromagnetic energy coupling were analyzed, and the electromagnetic susceptibility of sensors with different signal acquisition forms was identified.

### 3.1. Radiation Experiment Result

During the radiation test, the speed port and the power port of the main fuel regulation controller were monitored to obtain the coupling voltage and current signals of the system under the action of a strong electromagnetic pulse. By analyzing the voltage and current signals, the sensitive frequency points of the system were acquired.

**Figure 7:**
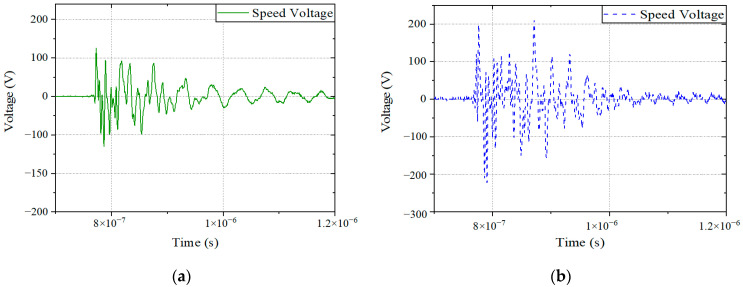
The time domain signal of the speed voltage: (**a**) Electric field strength is 10 kV/m; (**b**) Electric field strength is 20 kV/m.

**Figure 8:**
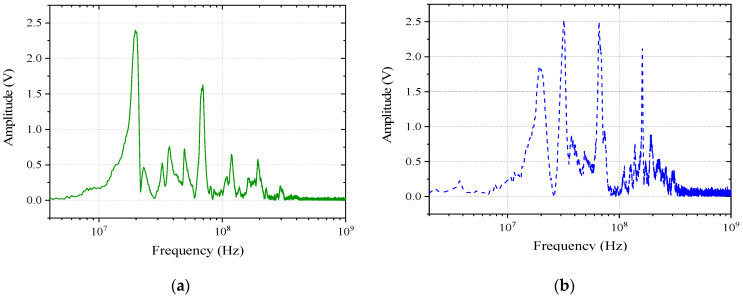
The frequency domain signal of the speed voltage: (**a**) Electric field strength is 10 kV/m; (**b**) Electric field strength is 20 kV/m.

The differential mode voltage signals of the speed port of the controller under different intensity vertical polarization electric fields are shown in Figure 7 and Figure 8, where Figure 7 shows the time domain signal of the differential mode voltage, and Figure 8 shows the frequency domain signal of the differential mode voltage. For the radiation test, the electric field intensity, the monitoring port voltage signal, and the main coupling frequency are listed in Table 2. The coupling frequency refers to the main resonant frequency of the monitoring port voltage signal. It is also the signal frequency corresponding to the resonant peak of the signal in Figure 8.

### 3.2. Pulse Current Injection Experiment Result

**Figure 9:**
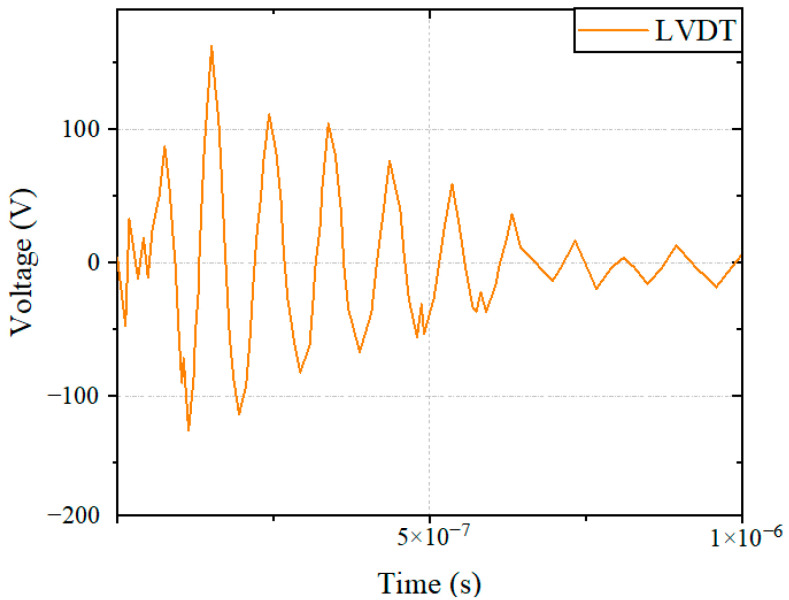
LVDT port monitoring voltage in 10 MHz current injection.

During the pulse current injection experiment, the voltage signals of the speed, displacement, and power port of the main fuel regulation controller were monitored to obtain the coupling signal of the system under the action of a strong electromagnetic pulse. By analyzing the voltage signal, the sensitivity threshold of the sensors was obtained. When a 10 MHz pulse current was injected into the LVDT connection beam, the LVDT port monitoring voltage of the controller is shown in Figure 9.

The main sensitivity phenomenon of the control system is the abnormal motion of the linear motor and the fluctuation of the DC motor. At the same time, the push rod of the linear motor is in a process from contraction to extension to normal, and the position signal appears abnormal. The speed of the DC motor changes from high speed to low speed or even stops, which is considerably larger than its normal fluctuation range. With the repeated injection of pulse current, the sensitivity phenomenon appears repeatedly. The displacement of the push rod of the linear motor and the speed of the DC motor repeatedly change.

**Figure 10:**
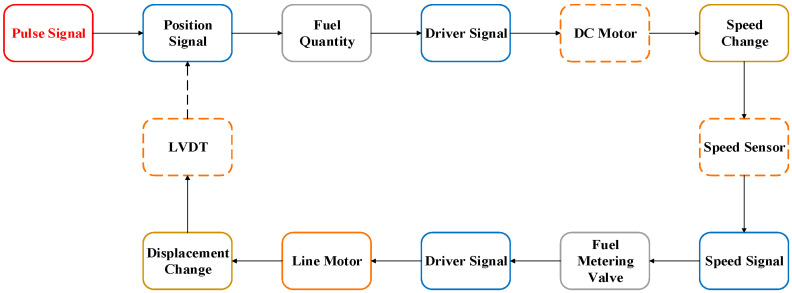
Interference path of the main fuel regulation control system.

To compare and analyze the electromagnetic pulse coupling of sensors, this paper analyzed the sensitivity of the LVDT sensor in the pulse current injection test. When the sensitivity phenomenon occurs, the differential mode voltage between the LVDT detection ports was 53.6 V. According to the control strategy of the main fuel regulation control system, the speed of the DC motor will increase, resulting in a change in the simulated engine speed collected by the speed sensor. In addition, the amplitude of the voltage signal increases. Simultaneously, to control the change in the engine speed, the system controls the output level of the linear motor, reduces the opening of the fuel pump valve needle, and reduces the amount of simulated fuel. Thus, the position of the simulated fuel pump valve needle changes, the displacement of the linear motor is shortened, the amplitude of the voltage signal collected by the LVDT is reduced so that the rotation speed of the DC motor is reduced, and the closed-loop control of the speed and displacement is completed, as shown in Figure 10. Through the analysis of the test results, the sensitivity threshold of the main fuel regulation control system under the action of a strong electromagnetic pulse was obtained.

### 3.3. The Sensor Susceptibility Law

**Figure 11:**
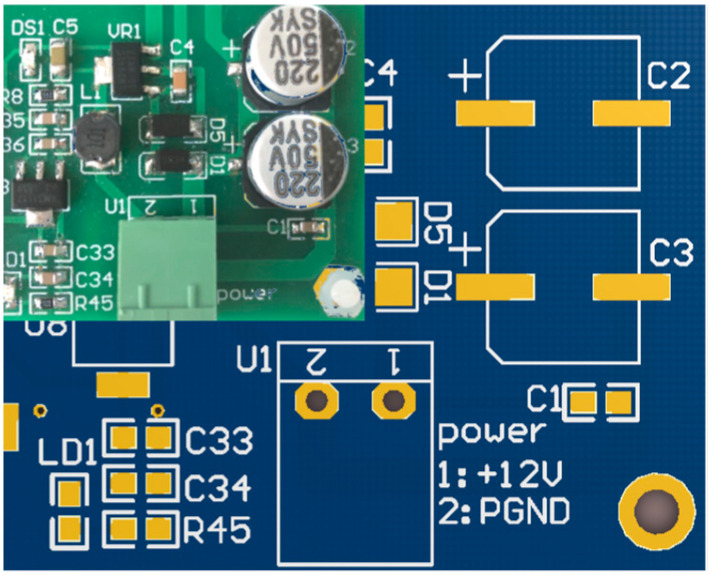
Power circuit of the main fuel regulation control system.

**Figure 12:**
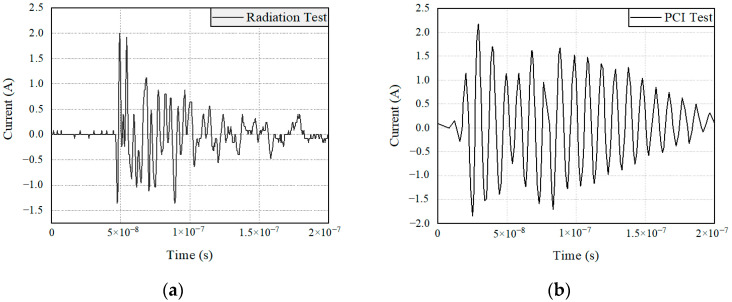
Power port signal: (**a**) In the radiation; (**b**) In current injection tests.

Usually, due to the different sources and mechanisms of electromagnetic pulses, the current distribution produced by radiation tests and pulse current injection tests on the test equipment is completely different. However, for the same controller terminal, if the input current of the monitoring port is equal, the two test methods can be considered equivalent. In this paper, the power port of the main fuel regulation control system was selected as the target. The electromagnetic sensitivity equivalence of the controller under two excitation modes was obtained by monitoring the current signal in the power port of the controller, as shown in Figure 11. The monitoring signal of the power port of the controller is shown in Figure 12.

In Figure 12, the current amplitudes of the radiation test and current injection test are 2 A and 2.17 A. The duration of the signals is 1.5 × 10^−7^ s and 1.7 × 10^−7^ s. The peak value of the signals decays by 90% at 1.3 × 10^−7^ s and 1.5 × 10^−7^ s. By comparing the current signals of the power monitoring port of the controller in the figure, it can be seen that the two test methods are equivalent to the electromagnetic sensitivity test results of the controller for the main fuel regulation control system.

**Figure 13:**
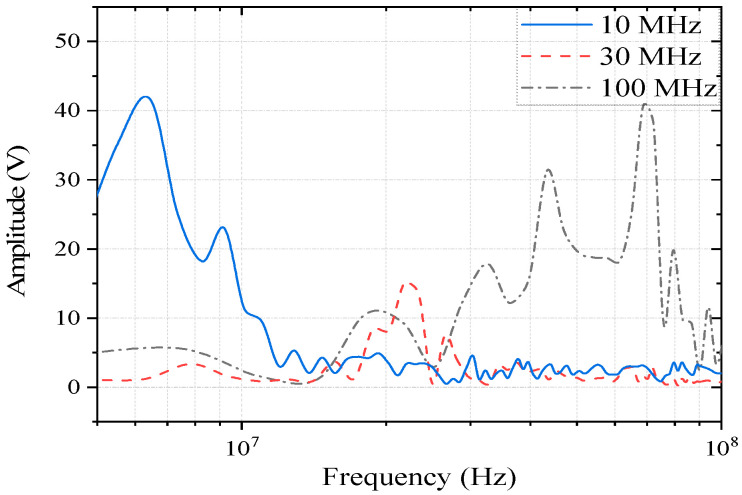
LVDT sensor terminal coupling voltage signal in the pulse current injection (PCI) test.

To explore the electromagnetic sensitivity characteristics of the speed sensor and LVDT sensor of the controller under the action of a strong electromagnetic pulse, pulse current injection tests were carried out on the two sensors based on the radiation test. The speed sensor controller terminal coupling voltage signal at the frequency domain is shown in Figure 7 and Figure 8. Based on the same current distribution of the controller terminal, pulse current signals of different frequencies are selected for injection into the system, and the voltage signal of the LVDT sensor port is monitored. The LVDT controller terminal coupling voltage signal at the frequency domain is shown in Figure 13. When we select the frequencies of the current signal at 10 kHz to 1 MHz to inject, the voltage signal of the LVDT sensor port is very small. The signal will not exhibit a distinct resonant frequency; it has no impact on the subsequent analysis of the sensors, so it is not shown in Figure 13.

The resonance frequency points of the terminal coupling signal in the speed sensor under different external excitations are basically consistent, and the main coupling frequencies are 20 MHz, 30 MHz, 70 MHz, and 160 MHz. The difference is that the coupling energy is different at each frequency. In the injection test, the monitoring signal of the controller terminal was analyzed, and the main coupling frequencies of the LVDT sensor under different external excitations were 6.5 MHz, 20 MHz, and 70 MHz.

**Figure 14:**
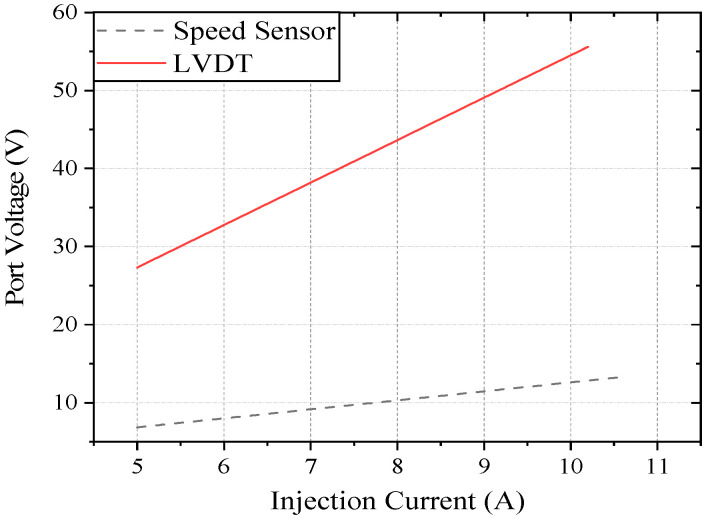
Speed sensor and LVDT terminal coupling voltage amplitude at the same frequency.

**Figure 15:**
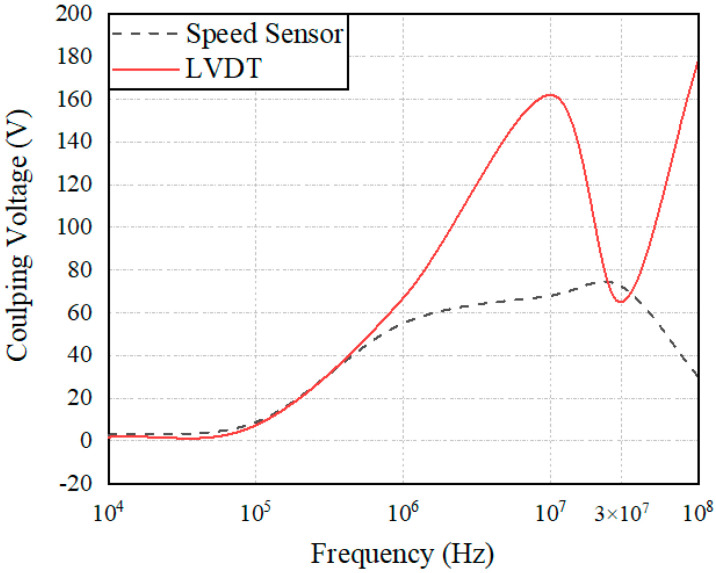
Speed sensor and LVDT terminal coupling voltage amplitude at the multifrequency.

A pulse current of 30 MHz was selected and injected into the connection beam of the speed sensor and the LVDT sensor to monitor the coupling voltage signal of the speed port and LVDT port of the controller, as shown in Figure 14. There is a large difference in the amplitude of the coupling signal collected by the two sensors under the impact of an electromagnetic pulse. To further examine the electromagnetic sensitivity of the two types of sensors under the action of a strong electromagnetic pulse, this study conducted multifrequency pulse current injection tests on the rotational speed sensor and the LVDT connection beam to monitor the amplitude of the coupling signal of the controller at 10 kHz, 100 kHz, 1 MHz, 10 MHz, 30 MHz, and 100 MHz. The curves were numerically fitted through six points, as shown in Figure 15.

**Figure 16:**
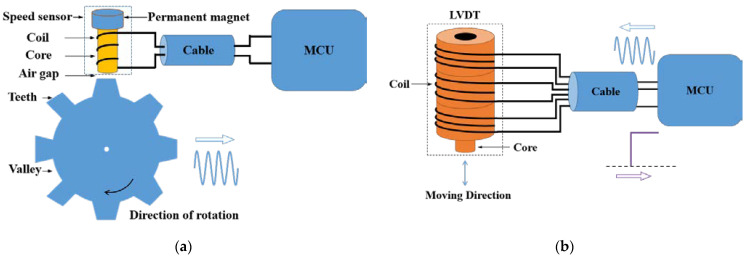
Working process of sensors: (**a**) Speed sensor; (**b**) LVDT sensor.

Compared with the curves in Figure 7, Figure 8, Figure 13 and Figure 15, the speed sensor and LVDT sensor differ concerning their sensitive frequency bands and their sensitivity threshold for the electromagnetic pulse. Referring to the sensitivity phenomenon in the test process, the LVDT sensitive band was 1 MHz to 10 MHz, and the speed sensor sensitive band was 30 MHz to 100 MHz. Based on the test results, the sensor coupling characteristics under different signal acquisition forms were obtained by analyzing the working principle and terminal circuit of the two sensors. A speed sensor is a kind of magnetoresistive sensor that produces induced current in the coil through the magnetoresistive change between the magnetic core and the phonic wheel. As an open magnetic circuit sensor, the LVDT sensor generates the induced current in the secondary coil through the position change of the moving magnetic core in the two-stage coil. The normal working flow of sensors is shown in Figure 16.

With the rotation of the phonic wheel, the speed sensor inputs a sinusoidal voltage signal to the controller of the main fuel regulation control system and transmits it to the single-chip microcomputer (MCU) through the signal filtering circuit. When the strong electromagnetic pulse is coupled to the speed sensor, the electromagnetic pulse enters the controller through the speed sensor connecting cable and forms a pulse voltage at the terminal. Similarly, with the change of the position of the main fuel metering valve, the LVDT sensor inputs a DC voltage signal after signal processing to the controller, which is transmitted to the single-chip microcomputer through the signal filtering circuit. When a strong electromagnetic pulse is coupled to the LVDT sensor, the electromagnetic pulse enters the controller through the connecting beam of the LVDT sensor and creates a pulsed voltage between the terminals.

**Figure 17:**
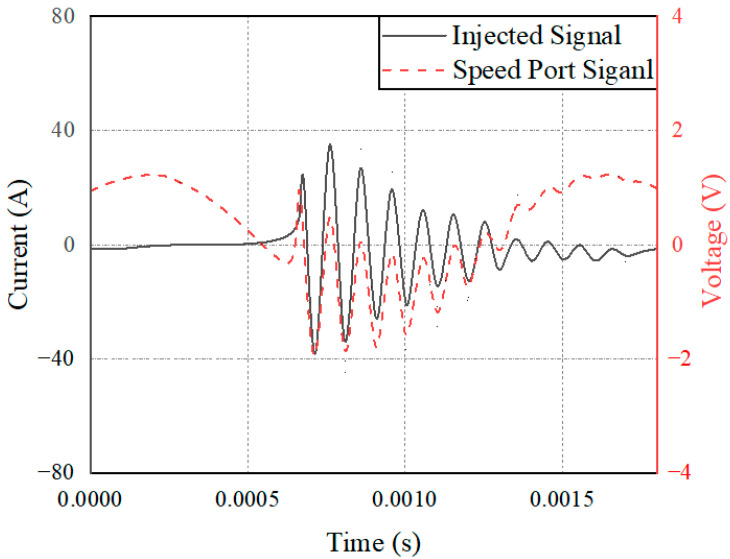
Speed sensor terminal coupling voltage signal.

**Figure 18:**
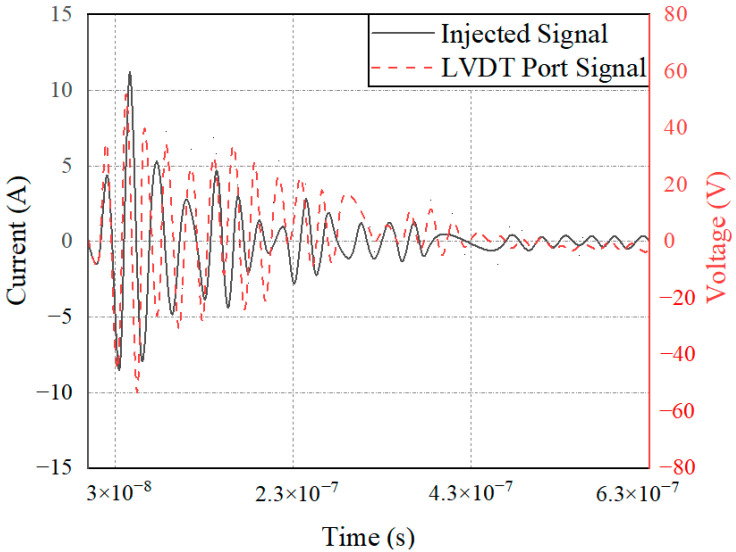
LVDT terminal coupling voltage signal.

However, the terminal circuits of the two sensors differ greatly in the strong electromagnetic pulse coupling effect, which is the reason for the difference in the electromagnetic sensitivity of the two sensors. On the one hand, the speed sensor is a passive sensor without signal excitation. In contrast, LVDT is an active sensor that needs to provide an excitation signal to excite. Therefore, when strong electromagnetic pulse coupling occurs in the sensor, the pulse will interfere with the LVDT excitation signal, resulting in LVDT excitation signal changes and sensitivity phenomena. On the other hand, the terminal circuit of the speed sensor identifies the input signal based on the zero-crossing detection method and calculates the current speed of the phonic wheel by obtaining the number of zero points. When the transient electromagnetic pulse couples into the terminal, the local voltage signal distorts, and the amplitude and waveform of the signal change, as shown in Figure 17. However, for the whole signal recognition cycle, the speed fluctuation over a very short time will not cause serious harm to the control system. The LVDT terminal circuit identifies the input signal of the sensor based on the quasi-linear relationship between the voltage signal and the position of the metering gate and calculates the current position of the gate by obtaining the signal level. When the strong electromagnetic pulse is coupled into the terminal, the terminal signal is distorted, and the amplitude changes, as shown in Figure 18. Moreover, due to the linear relationship between the level and the valve position, the position obtained by the control system is misjudged. Under the action of the main fuel control strategy, incorrect instructions are issued to the fuel pump, resulting in relatively poor results for the control system.

By analyzing the amplitude and frequency of the coupling signal of the cable terminal in the BCI test, it is concluded that the coupling signal is the linear superposition of the pulse injection signal and the sensor detection signal in the time domain. However, in the speed signal detection period, the coupling signal has little influence on the speed signal of the control system and will not cause the electromagnetic sensitivity of the control system. In the displacement signal detection period, the coupling signal has a great influence on the change of the displacement signal of the control system, thus causing the electromagnetic sensitivity of the control system.

In general, the two sensors based on the principle of electromagnetic induction will couple higher voltage at the controller under the action of a strong electromagnetic pulse, which will cause interference to the main fuel regulation control system. However, due to different forms of action, the electromagnetic pulse affects them quite differently, which is why the LVDT is sensitive during the injection test and the speed sensor is not.

## 4. Conclusions

Based on the electromagnetic coupling effect of the main fuel regulation control system, this study analyzed the equivalence of the radiation test method and the current injection test method. The sensitivity thresholds of the sensors under the action of the force electromagnetic pulse were obtained. The sensitive frequency band of the LVDT sensor was 1 MHz to 10 MHz. The sensitive frequency band of the speed sensor was 30 MHz to 100 MHz. We analyzed the interference effect of sensors on the control system. The signal at the same monitoring port in the controller of the main fuel regulating control system was compared and analyzed under two test methods. The results were consistent with respect to amplitude and the main coupling frequency. The above method can prove the equivalence of the two methods for determining the terminal electromagnetic pulse effect on the controller.

In addition, based on the structure, working principle, and terminal circuit analysis of the sensors, the electromagnetic effects of two different sensors under the action of a strong electromagnetic pulse were obtained. Scholars can study the sensors’ coupling mechanism by the electromagnetic coupling effect of the control system under a strong electromagnetic pulse. It provides a basis for the measurement technology and sensor design improvement of the control system and lays a foundation for electromagnetic pulse protection of digital systems. In the future, the authors will conduct electromagnetic pulse sensitivity tests on an actual aero-engine control system to obtain the electromagnetic effects of more aircraft under the action of a strong electromagnetic pulse and to improve the safety of aero-engines in complex electromagnetic environments.

## Figures and Tables

**Table 1:** Parameters for Equation.

Symbol	Quantity	Value
*E*	Electric field strength	10,000 to 50,000 V/m
*k*	Waveform factor	1.3
*α*	Attenuation coefficient	4 × 10^7^ s^−1^
*β*	Phase shift coefficient	6 × 10^8^ s^−1^
*I*	Injection current strength	5 A; 10 A
*f*	The frequency of the injecting current	10 kHz to 100 MHz
*σ*	Damping coefficient	Based on the frequency

**Table 2:** Speed Sensor Coupling Voltage Signal.

Electric Field Intensity	Voltage Peak	Coupling Frequencies
10 kV/m	126 V	20 MHz and 70 MHz
20 kV/m	210 V	20 MHz, 30 MHz, 70 MHz and 160 MHz

## Data Availability

Data are available on request to the authors.
